# Characterization, Properties and Mixing Mechanism of Rubber Asphalt Colloid for Sustainable Infrastructure

**DOI:** 10.3390/polym14204429

**Published:** 2022-10-20

**Authors:** Lu Zhang, Chuanping Zhang, Zhen Zhang, Hanbing Wang, Shifeng Wang

**Affiliations:** 1 School of Chemistry and Chemical Engineering, Shanghai Jiao Tong University, Shanghai 200240, China; 2 China Construction Infrastructure Corp., Ltd., Beijing 100029, China

**Keywords:** devulcanized rubber, rubberized asphalt, colloidal structure, interactions, mixing mechanism, sustainability

## Abstract

Rubber asphalt has always been considered to have the most potential for the disposal of waste tires as sustainable infrastructure. However, the covalently cross-linked tire rubber presents an extreme challenge in reusing waste rubbers in roads. Rubberized asphalt with finely dispersed or colloidal structure has been regarded as a potential binder used as road material because of the improved properties in terms of storage stability, easy processing and high content of incorporation. However, the mixing mechanism between the finely dispersed rubber on micro-nano scale with asphalt is still not clear, which restricts its further development as value-added material. Devulcanized rubber (DR) was introduced to improve the compatibility between asphalt and rubber. The basic chemicals of DR and asphalt were introduced based on their structures. Furthermore, the interactions between DR and asphalt were discussed according to the functional elements at different levels, and the concept of DR as “the fifth component” of asphalt was put forward. Finally, high performance, environmental and economic effects and applications of devulcanized-rubber-modified asphalt (DRMA) were discussed. The review is expected to provide a guide for the wide application of DRMA, which is still restricted by poor compatibility and bad stability during processing, storage and recycling.

## 1. Introduction

One of the key challenges in waste management, especially in the disposal of end-of-life tires, has always been the task of monitoring in the 21st century [1]. The most widespread disposal methods for end-of-life tires are landfills and incineration [2], causing much environmental pollution due to the nonbiodegradability, flammability and chemical characteristic of scrap tires, such as the emission of toxic gases and other harmful substances [3,4]. On the other hand, scrap tires are mainly composed of vulcanized rubber, reinforcing filler and a variety of materials, which is not only an environmental pollutant but also a great economic loss. Therefore, recycling and reuse of these waste materials are of great significance considering the world is facing a lack of resources and a shortage of energy.

The use of ground tire rubber (GTR) for modifying asphalt is very promising and is a sustainable development strategy [5]. The addition of GTR to asphalt shows many improvements in the physical, chemical and mechanical properties of the rubber asphalt binder, such as enhanced stiffness, increased skid resistance, extended service life, mitigated fatigue cracking and so on [6,7,8]. However, the multiple cross-linking network structure of GTR leads to not only high energy consumption and pollution emissions but also results in the poor compatibility and bad stability of rubber asphalt. The above problems limit the efficient utilization and cause the unscientific application of rubber asphalt. In view of this, devulcanization or degradation of the cross-linking network is urgent for reutilization with high efficiency.

**Figure 1:**
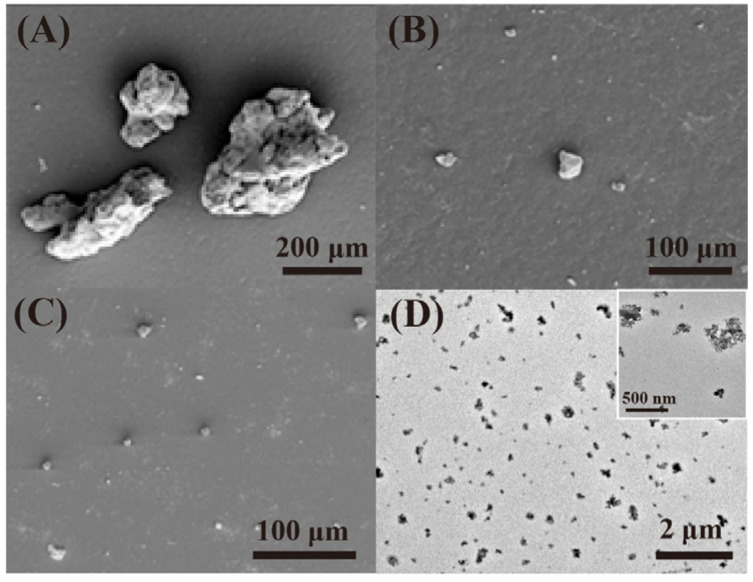
(**A**–**C**) SEM micrographs of carbon black obtained from (**A**–**C**) at different degraded temperatures of 260 °C, 280 °C and 300 °C [10]; (**D**) TEM micrograph of degraded rubber in the asphalt [12]. Insert: The enlarged TEM micrograph.

In order to obtain stable rubber asphalt with good performance, the compatibility between asphalt and rubber should be improved. Tire rubber is usually composed of cross-linked rubber, carbon black (CB), silica and so on [9]. Devulcanization or degradation destroys the cross-linked network and rubber body carbon chains to be short chains and other separated small substances such as CB. Most previous studies focused on the devulcanization of rubber and the reduced molecular weight of sol fraction (Table 1); less is reported about the decreased size of CB (from several micrometers to micro-nanometers, Figure 1A–C) with the increase in degradation degree [10,11]. These degraded substances are well dispersed in the asphalt, as shown in Figure 1D [12]. Therefore, deeply degraded rubber provides a new strategy for preparing rubberized asphalt from a different perspective.

The DR mainly consisted of micro-nano CB, low molecular weight of sol fraction and inorganic substances, denoted as micro-nano rubber. The DR showed much better dispersibility and reinforcement compared with GTR, thus usually used for the modification of asphalt with high content. Therefore, the DR was more and more used as the potential alternative for asphalt in pavement, which significantly improved the anti-cracking and anti-aging performance [13,14,15]. Although DRMA demonstrates much improved properties compared with both base asphalt or GTR modified asphalt, the standardized applications of rubber asphalt have always been a challenge for the lack of a deep understanding of the mixing mechanism, especially on a colloidal scale.

Until now, there is no strict classification for the details of the reaction between DR and asphalt. In order to further guide the application of rubberized asphalt by theory, this paper first reviewed the chemical composition of DR and asphalt, summarized the colloidal structure and mixing mechanism of DR with asphalt and discussed the interaction between CB, ZnO and other additives in micro-nano tire rubber and different fractions of asphalt. Finally, the sustainability of rubberized asphalt was evaluated, including the improved performance, economic benefit and environmental effects. Importantly, a meta-analysis of emissions for rubber asphalt was performed, offering an objective deduction. This review will provide basic theoretical support for the application of rubber asphalt.

## 2. Tire Rubber and Its Degradation Behavior

### 2.1. Chemical Composition of Tire Rubber

Tire rubber has a crucial role in many fields such as industry, agriculture and national defense. It is reported that the global tire output is about 1.5 billion each year and that of waste tires is more than 17 million tons each year. With the increased demand for tires in developing countries, the output of waste tires grows more rapidly. Due to the cross-linking and reinforcing network structure of waste tires, they are difficult to degrade under natural conditions, which results in a large accumulation. Serious “black pollution” formed by wasted rubber severely threatens the health and life of human beings.

**Figure 2:**
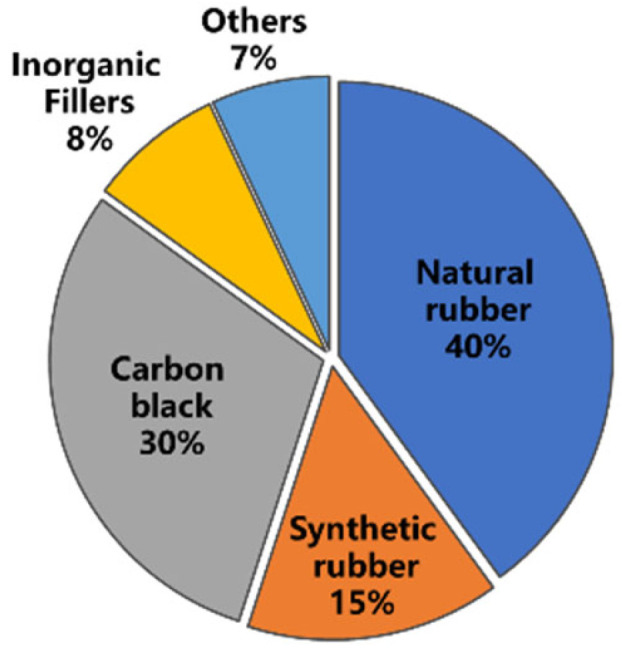
The composition of the GTR from truck tire [16].

Tires mainly consist of vulcanized rubber and a series of additives [16]. The composition of a typical tire is shown in Figure 2 (by weight). It is mainly composed of natural rubber (NR) which comprises about 40% of the weight of truck tire rubber, making the tire full of elasticity and durability even under heavy loads [17,18]. The proportion of NR has increased nowadays, possibly due to the expansion in the use of radial tires and heavy-duty tires. It should be pointed out that the properties of NR can largely affect the workability of materials. For example, the molecular weight and gel content of NR may affect compound viscosity and filler dispersion in rubber mixing. In addition, the tendency of NR to crystallize upon stretching, even before cross-linking, has contributed to the strength of uncured rubber compounds.

Adding fillers to NR can improve interfacial adhesion and promote uniform dispersion, thus enhancing a series of properties of products, including stiffness, hardness, wear resistance, etc. Nanostructured CB and silica (SiO_2_) have a large surface area, allowing them to be dispersed in the rubber on a microscale.

CB is a proven reinforcing filler and has been widely applied in rubber, due to the improvement of mechanical properties, aging resistance, etc. Ayippadath et al. reported a more than 10-fold increase in tensile strength for SBR after adding N330 CB filler [19]. However, CB from petroleum is non-renewable and contaminative; therefore, recycling and reusing CB are quite necessary [20]. Silica is another widely used reinforcing filler. Different from CB, it is independent of oil resources and shows great advantages in low rolling resistance [21]. Idrus et al. evaluated the functions SiO_2_ plays in vulcanized NR [22]. They proved that irregularly shaped silica with ultra-fine particle size can better improve torque values. Many other chemicals are also used in rubber, such as cross-linking agents, antioxidants, plasticizers, etc. [23].

Different tires have distinguishable compositions; even different parts of the whole tire are different. However, their basic formulations and main components do not vary much: 50% rubber and 30% filler, as far as we know.

### 2.2. Degradation Behavior of Tire Rubber

Due to the irreversible vulcanization of tire rubber, conventional GTR with three-dimensional chemical cross-linking is an insoluble and infusible thermoset material, making it difficult to recycle and reuse. Therefore, the reclamation of vulcanized rubbers is urgent.

Reclamation is a procedure where the vulcanized rubber is converted into a product that can be vulcanized, processed and mixed again by using thermal energy and chemicals. The mechanism of this process is devulcanization or degradation [24]. In degradation, the breakage of chemical bonds occurs in the network, such as the C-S bond and S-S bond, and the chains of rubber are shortened as a result. While for devulcanization, the C-S bond, S-S bond and C-C bond are all broken without the scission of the main chains of rubber. Once the reclamation is increased seriously, the CB will separate from the rubber, and lightly pyrolyzed tire rubber will occur.

**Figure 3:**
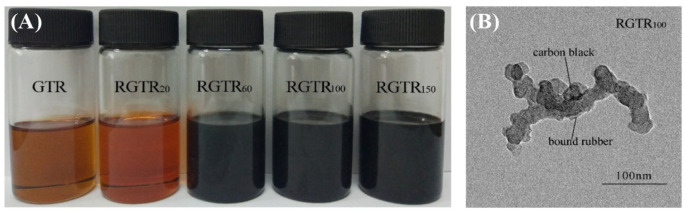
(**A**) Sol fractions obtained from degraded rubber with types of soybean oil under 150 °C for 4 h; (**B**) TEM image of CB from RGTR_100_ [26].

Song et al. reported the sol fractions and core–shell CB nanoparticles (Figure 3) in the process of reactive extrusion or warm aging [25]. They also reported a heterogeneous degradation of covalently cross-linked networks of GTR to core–shell CB nanoparticles [26]. Li et al. achieved the separation of CB from GTR by melt-extrusion pyrolysis at 300 °C [10]. Terminal blend technology can induce devulcanization and degradation of GTR at a high temperature of over 260 °C in asphalt but gives off a huge fume [11]. Seghar et al. summarized the previous works of rubber reclamation, desulfurization and regeneration for GTR in detail [18,27,28,29].

**Figure 4:**
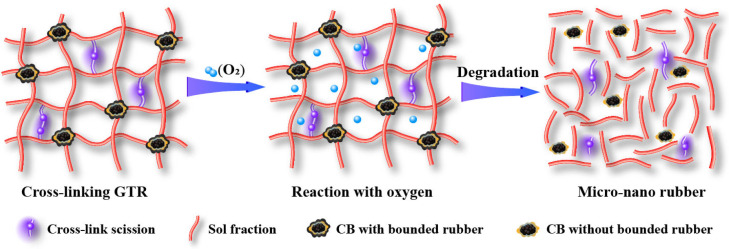
Schematic diagram of the dynamic thermal-oxidative reclaiming process [30].

Wang et al. developed a new method named “thermal-oxidative reclamation” for devulcanizing GTR (Figure 4) [30]. This environmentally friendly method induces the completed breakage of the cross-link and part breakage of the main chain, leading to a decreased molecular weight of the sol with high content. The CB with micro-nano size can be released from the network of GTR.

**Figure 5:**
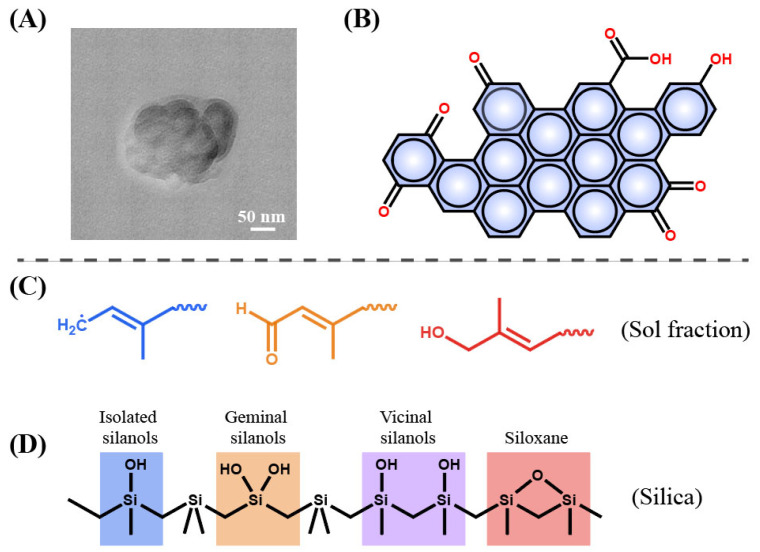
Components of DR. (**A**) TEM micrographs of CB [25]; (**B**) surface chemistry of CB [31]; (**C**) surface chemistry of silica [30]; (**D**) chemical formula of low molecular sol fraction [32].

The DR produced by the light pyrolysis process mainly consists of CB, sol fraction and inorganic species such as ZnO, silica, ZnS and so on. The CB has a very thin layer of tightly bound rubber, resulting in a core–shell structure (Figure 5A) [25]. With a crystal morphology between graphene crystal and amorphous, CB is usually less than 10 μm in diameter. Importantly, many polar functional groups including hydroxyl (-OH), carboxyl (-COOH), and amino groups (-NH_2_) are exposed on the surface of CB (Figure 5B), which provides an active site for the chemical reaction with asphalt [31]. In addition, sol fraction shows a relatively low molecular weight and is expected to be an alternative to asphalt because of its asphalt-like properties (Figure 5C) [30]. The inorganic products mainly contain ZnO, silica and ZnS. Silica is mainly composed of SiO_2_, and it can be expressed as SiO_2_·nH_2_O. nH_2_O in the SiO_2_·nH_2_O formula exists in the form of isolated silanols, vicinal silanols and geminal silanols (Figure 5D) [32]. These hydroxyls in silanols provide the possibility of intermolecular chemical reaction with other species such as asphalt.

Previous reports found that DR could significantly improve the crack resistance of asphalt, providing an important foundation for the application and analysis of rubber asphalt [33].

## 3. Colloidal Structure of Asphalt

**Figure 6:**
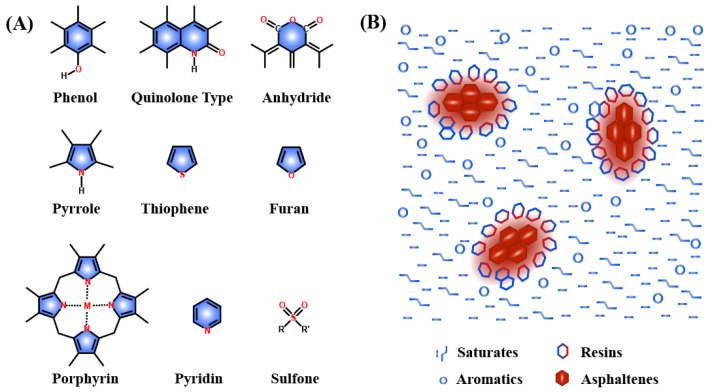
(**A**) Typical functional groups present in asphalt; (**B**) colloidal structure of asphalt [34].

Asphalt has been used as a construction material for hundreds of years. Usually, the density of asphalt ranges from 1.01 to 1.04 g/cm^3^ at room temperature [34]. Generally, asphalt mainly consists of the C element (ca. 80–88%), H element (ca. 8–12%), S element (<10%), O element (<2%) and nitrogen element (<2%) by weight. The hydrocarbon content is over 90 wt.% with a H/C ratio of around 1.5. In addition to the above elements, traces of vanadium, iron, nickel and manganese are also found. The above elements existed in asphalt in the forms of phenol, anhydride, pyridine, porphyrin thiophene, etc. (Figure 6A). Asphalt is widely accepted to exist as a colloidal structure, in which asphaltenes form the core, polar naphthene aromatics are considered to be micelles, and they are evenly dispersed in saturates (Figure 6B).

Separation of asphalt into four polarity-based fractions was developed and reported by Corbett in 1969 [35]. Usually, the separation can be achieved through the chromatographic method by using solvents with different polarities such as the heptane-toluene-ethanol system. After the separation, saturates, aromatics, resins and asphaltenes are collected, with the distinguishing chemical composites summarized in Table 2.

Saturates, with a H/C ratio of ca. 2, typically amount to 5–15% of the whole fraction. The number-average molecular weight of saturates is ~600 g/mol because saturates are mainly composed of nonpolar alkanes and aliphatic cycloalkanes.

Aromatics are mainly composed of unsaturated cyclic aromatic hydrocarbons. They can show some polarity when nitrogen and sulfur heteroatoms exist. Aromatics are usually a liquid with a yellow to red color under room temperature. Compared with saturates, the viscosity and density of aromatics are somewhat higher, possibly owing to the larger average molecular weight (~800 g/mol) and higher glass transition temperature. Notably, the percentage content of N and S elements in aromatics might be as high as 4%.

Resins are one fraction with a state between solid and liquid. They show deeper color, greater polarity, larger molecular weight and higher density when compared with saturates and aromatics. Generally, resins can transform into asphaltenes when they are overexposed to air, due to poor chemical stability. In addition, resins play an important role in stabilizing the colloidal nature of asphalt as a result of the saturated and aromatic characteristics [36].

Asphaltenes in the form of black solids are soluble in benzene but not in n-pentane. Similar to resins, asphaltenes are mainly composed of polyaromatic and heterocyclic aromatic rings. Asphaltenes are components that contain numerous inorganic components, including N, O and S heteroatoms and metal ions such as Fe, V and Ni. With high molecular weight, asphaltenes are brittle and hard and may precipitate during production, refining, transportation and storage.

## 4. Mixing Mechanism of DR with Asphalt

Rubberized asphalt has been successfully applied in pavement construction and rehabilitation for many years. The traditional wet process employed GTR as an asphalt binder modifier by mixing it at 180–190 °C to generate rubber asphalt. This technique significantly improved the engineering performance of pavement compared to the conventional method, but the poor storage stability and dense compaction are of great concern due to the formation of a solid–liquid two-phase system. Afterward, terminal blend mixing technology arose in America. This process degraded GTR into small-molecule polymers in asphalt at high temperature, high speed and long-time shearing, thus improving storage stability and workability [37,38,39]. It was found that DR with micro-nano size could mix asphalt more quickly and completely. This is mainly because the scission of both the main chain and cross-link network in degradation mediated the formation of smaller products compared to those obtained from degradation. The colloid structure and mixing mechanism between DR and asphalt are analyzed as follows.

### 4.1. Distribution of Typical Elements and Functional Groups

Elements such as C, S, N and O are abundant in rubber. The distribution and content of these elements will greatly affect the rheology, aging resistance and temperature sensitivity of rubber asphalt. Since the sol fraction of micro-nano rubber composed mainly of alkynes and alkenes shows small polarity, it is easily dissolved into saturates and aromatics of asphalt, leading to the entry of a small amount of S and O elements as they partly exist in broken cross-links. Meanwhile, the surface of CB particles, the key product of DR, is rich in functional groups, including -OH, -NH_2_, -SH, -COOH and -SO_2_-, etc. These groups with great polarity tend to repulse saturates and aromatics on one hand. On the other hand, complex physicochemical interactions with resins or asphaltenes are expected because they are all full of various groups. As a result, N, O and S elements in resins and asphaltenes show an increase after mixing. Since these elements are closely related to the aging or anti-aging properties, the increased elements are likely to be an important factor that affects the aging resistance of rubber asphalt [40]. Additionally, the entry of oxygen-containing groups (e.g., -OH and -COOH) changes the interaction between the rubberized asphalt and limestone powder.

Meanwhile, tire additives such as antioxidants, anti-aging agents, cross-linking agents and accelerators in DR containing S, Zn and Si elements are mostly inorganic and tend to combine with asphaltenes. Although the amount of these elements is quite small, they may bring significant improvement in performance including anti-aging, anti-oxidation, etc.

### 4.2. Interactions between DR and Asphalt

**Figure 7:**
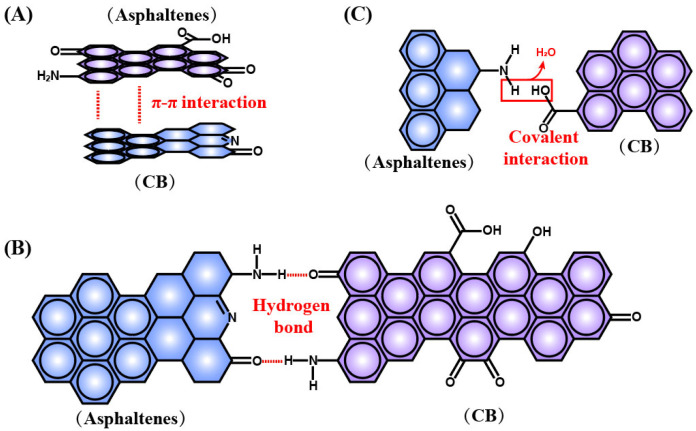
(**A**) Typical interactions between DR and asphalt. (**A**) π-π nonbonding intermolecular interactions; (**B**) intermolecular hydrogen bonds; (**C**) covalent interaction.

The DR can efficiently and completely interact with different components of asphalt. Firstly, the sol fraction in DR and light components in asphalt are both less/nonpolar, and thus they are easy to directly penetrate and dissolve into each other, with little chemical reaction. Differently, there are many types of interactions between polar-functional-group-containing CB, silica, resins and asphaltenes. The graphene crystal-like structure of CB and polycyclic aromatic hydrocarbons of resins and asphaltenes provide effect conditions for π-π nonbonding intermolecular interactions between CB and resins/asphaltenes due to abundant conjugated structures (Figure 7A).

The multiplex functional groups also make it possible for the above species to exhibit positive or negative electricity, leading to the electrostatic interaction between DR and asphalt, such as the interaction between the N atom in the -NH_2_ group from the CB surface and O atom in the -OH group from asphaltenes. Intermolecular hydrogen bonds can also be observed between DR and asphalt (Figure 7B), especially between CB and asphaltenes, owing to the existence of large numbers of -NH_2_ groups and -OH groups. The silica is also abundant with -OH in the form of isolated silanols, vicinal silanols and geminal silanols, thus combining with asphaltenes by considerable hydrogen bonds, too.

In addition to the intermolecular weak interaction, covalent interaction is the direct evidence that DR chemically reacts with asphalt (Figure 7C). There are many chemical reactions by generating covalent bonds between CB and resins/asphaltenes, including condensation reaction between carboxyl and amino, nucleophilic addition between phenolic aldehyde and carbonyl, addition reaction between alkene and amino catalyzed by alkali metal or alkaline-earth metal from asphaltenes, coordination reaction between porphyrins and N-containing functional groups, reversible fracture recombination of polysulfide bonds, etc. These reactions make it possible to mix DR and asphalt through strong chemical interaction, which largely improves the compatibility and stability of DRMA and may mediate some behavior such as self-healing [41].

Furthermore, owing to the existence of crystals in DR or asphalt, such as graphite crystal and wax crystal, doping of other ions into crystal defect may be observed in the mixing process. However, the effect of crystal structures on the asphalt performance is still not clear, so the doping behavior in the mixing of DR and asphalt should be further studied.

In short, both physical and chemical interactions exist between DR and asphalt. The complex interactions should be classified and analyzed according to the specific reaction types to deeply understand the influence of different interactions on the structure and performance of rubber asphalt.

### 4.3. Compatibility of DR and Asphalt

The compatibility of rubber and asphalt fundamentally determines the performance and stability of the modified asphalt [42]. Segregation during transportation, storage and construction must be avoided in order to prepare roads with desired properties. Unfortunately, vulcanized rubber and asphalt are poorly compatible. [43].

Once the vulcanized network structure was lightly pyrolyzed, the soluble rubber and micro-nano carbon black were released. According to the second thermodynamic mixing rule, the strong interactions among soluble rubber, carbon black and asphalt are the most important factors that affect the compatibility [44]. Secondly, the entropy plays an important role, as the mixing entropy obviously increased as the low molecular weight of liquid rubber.

(1) $$\Delta G_{m}=\Delta H_{m}-T\Delta S_{m}$$

where *G*, *H*, *T* and *S* represent Gibbs free energy, enthalpy, temperature and entropy. Solubility parameters are a good assessment for evaluating the compatibility between different substances [45]. The solubility parameters between DR and asphalt are similar, so they are easy to dissolve into each other, which is not available in GTR-modified asphalt.

**Figure 8:**
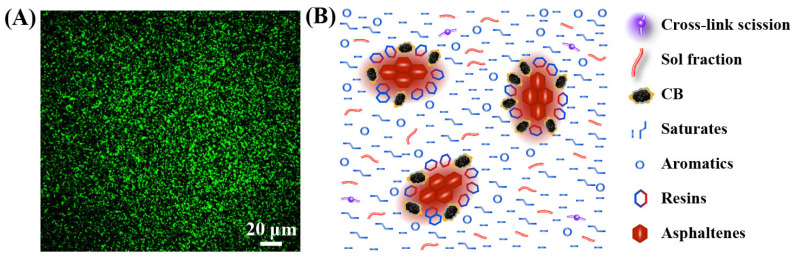
(**A**) Typical morphology of DRMA observed by fluorescence microscopy [46]; (**B**) colloidal structure of micro-nano rubber asphalt.

To the best of our knowledge, methods such as microscopic observation, segregation tests and interaction measurement are usually used for evaluating the compatibility between rubber and asphalt. Tang observed that the DR was well dispersed in asphalt at the nanometer or micrometer scale, which reflected the improved compatibility between DR and asphalt (Figure 8A) [46].

### 4.4. Colloidal Structure of DRMA

From the perspective of morphology, asphalt stabilizes itself in the form of colloidal structure. After mixing, the sol fraction of DR dissolves in saturates and aromatics to form clusters as a new continuous phase (the medium of suspension) of rubber asphalt (Figure 8B). This phase not only makes the morphology more compact for avoiding the invasion of water and oxygen but also reduces the volatilization of light components in the aging process. Therefore, the DRMA is excellent in anti-aging and waterproofing [47].

Moreover, CB particles are combined with asphaltenes to form a new dispersed phase (the suspended particles) of rubber asphalt colloid. Due to the removal of most bound rubber onto the surface of CB, CB is able to have a small size, avoiding the segregation and sedimentation in asphalt on one hand. And on the other hand, the decrease in bound rubber from CB weakens the oil absorption ability, so DRMA can be obtained with a high content of DR and has a relatively low viscosity (50% content, 135 °C viscosity < 3 Pa/s). Except for CB, inorganic substances such as ZnO and ZnS will combine with CB and asphaltene as suspended particles, bringing special performance.

The resins in the base asphalt, as the peptizator, are the key component to maintain the stability of the asphalt colloidal structure. Usually, resins distribute around asphaltenes and prevent asphaltenes from precipitation. In DRMA, resins largely remain relatively the same as in the previous base asphalt colloidal system. Differently, resins surround the asphaltenes, carbon black and inorganic salts, but not original asphaltenes anymore, to maintain the stability of DRMA.

Compared with traditional GTR with hundreds of microns of CB, DR with small size and low density is less oil consuming and is expected to become “the fifth component” of asphalt. After mixing, DR can directly, rapidly and completely react with asphalt to form a new colloidal structure with no precipitate, providing a new strategy for constructing pavements with high performance.

## 5. Sustainability of Rubberized Asphalt

**Figure 9:**
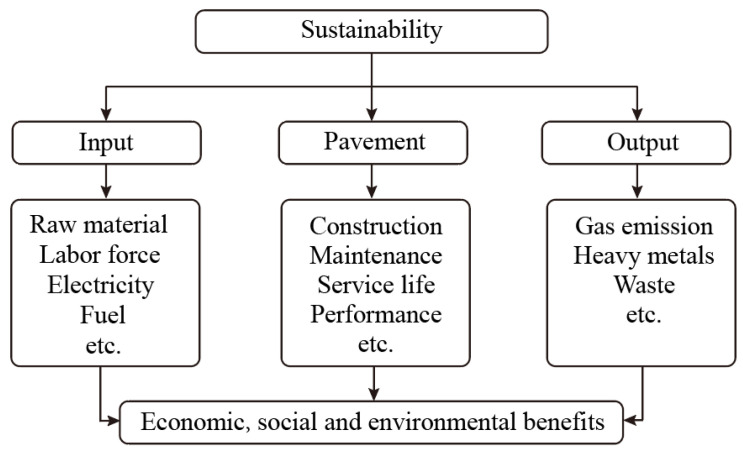
A typical framework for assessing the sustainability of an asphalt pavement.

The sustainability of a rubber asphalt pavement should take into account the following three aspects: input, pavement service process and output (Figure 9). Each stage of these aspects, including raw material, labor force, electricity, service life, performance and emissions to air/water, has significant effects on the economy, health and environment [48].

### 5.1. Performance for Rubber Asphalt Pavement

DRMA shows improved properties, such as mechanical and rheological properties, aging resistance, rutting resistance, stability, moisture susceptibility, temperature sensitivity and durability, compared with traditional GTR-modified asphalt or base asphalt [49,50,51,52,53].

The lower glass transition temperature of DR brings good deformability and elasticity even under −30 °C [54]. On the other hand, DR acting as a stress concentrator in the mixing system can avoid the generation of low-temperature cracks and inhibit the crack expansions for pavements.

The weak bonding energy between vulcanized rubber and asphalt usually leads to segregation. For DR, the cross-link bonds are broken, and linear molecules are released from the network, thus decreasing the size of rubber. Such a method improves compatibility and avoids phase separation.

The excellent aging resistance of rubberized asphalt is widely accepted [55,56,57]. The improvement of aging resistance is mainly due to three reasons. Firstly, the asphalt film thickness and viscosity of DRMA are higher than those of base asphalt, avoiding the exposure of asphalt to air. Secondly, the release of carbon black from the cross-linked network can bind with asphaltene, preventing the oxidation of asphalt from UV light and oxygen [56]. In addition, the cross-linking reaction that happened to carbon–carbon double bonds and sulfur-containing bonds brought about a self-healing effect to DRMA, increased fatigue and low-temperature crack resistance [58].

### 5.2. Emissions and Environmental Effects

Global energy scarcity is one of the most serious sustainable challenges for the international community. Circular economy and emission reduction are the keys to alleviating this problem. The application of DR in asphalt pavements greatly solves the problem of disposal of waste tires, which brings outstanding economic and environmental benefits due to the reduced use of raw materials and the extended service life of pavements [59].

Studies based on life cycle assessment (LCA) indicated that the life cycle of pavements mainly contains raw material manufacturing, construction, service, maintenance and end of life. DRMA significantly saves energy in each of these processes. [60] The preparation of DRMA does not need very high mixing and compaction temperatures compared with traditional hot mix asphalt, generating less greenhouse gas emissions and lower costs. Moreover, the enhanced mechanical properties of DRMA pavement make fewer cracks and little maintenance possible, saving a lot of energy and natural resources. Until now, DRMA has been very attractive in the road industry.

The previous studies concluded that the longer service life is one of the main factors that reduce emissions and energy consumption [61]. The life of a properly designed and constructed rubber asphalt pavement can be more than 20 years, which is twice that of conventional asphalt pavement. Liang et al. proved a higher energy consumption for DRMA than that of SBS-modified asphalt, possibly owing to the production of an SBS modifier [62]. Actually, Farina et al. calculated that the energy saving of crumb rubber was as high as 4236 MJ/t, which reduced the energy consumption for materials containing rubber [63]. Theoretically, DRMA will save more energy.

Pollutants during the life cycle of asphalt pavements are mainly released into the air and water. Kucukvar et al. found that the production and transportation of materials were two main processes that generated atmospheric emissions [64].

Fumes from asphalt pavement construction are a potential source of adverse environmental effects on human health. Usually, the fumes consist of polyaromatic hydrocarbons (PAHs) and volatile organic compounds (VOCs) [65]. However, it seems that the fumes are mainly generated from asphalt. We also retrieved the literature and performed a “meta-analysis” to find out whether rubber asphalt increases emissions.

As shown in Figure 1, the relevant peer-reviewed journal articles were searched through Pubmed and Embase [66,67,68,69,70,71,72]. The search expressions involved “rubber asphalt” and “emission” or “pollution” or “economic” or “energy consumption”. Essential information was extracted from each article for further analysis. We carried out two “meta-analyses”: association between PAH/VOC emissions and rubber asphalt, and association between benzothiazole and rubber asphalt.

**Figure 10:**
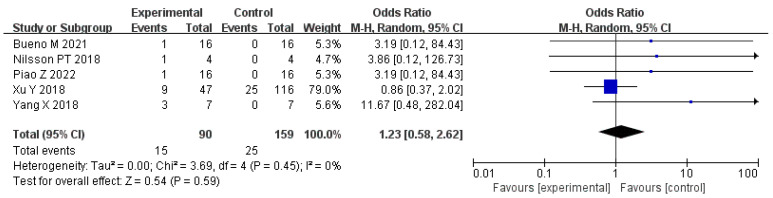
Meta-analysis of PAHs/VOCs and rubber asphalt. The solid horizontal lines indicate the 95% CI. The size of blue squares indicates weight of study in the analysis. The black diamond with indicates the pooled odds ratio estimate, with 95% CI represented by the diamond width.

**Figure 11:**
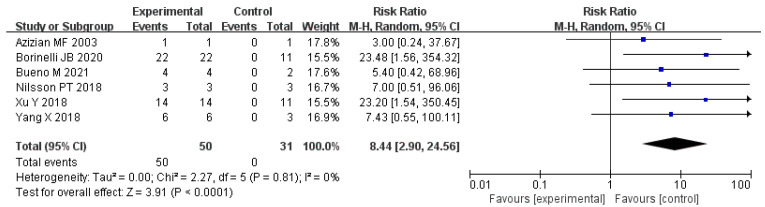
Meta-analysis of benzothiazole and rubber asphalt. The solid horizontal lines indicate the 95% CI. The size of blue squares indicates weight of study in the analysis. The black diamond with indicates the pooled odds ratio estimate, with 95% CI represented by the diamond width.

After excluding lower-quality studies, five journal articles were selected for meta-analysis in terms of the association between PAH/VOC emissions. The meta-analysis did not show indication of heterogeneity (*p* = 0.45, I^2^ = 0%), and meta-RR = 1.23 (95% CI 0.58 to 2.62) (Figure 10). No increase in PAH/VOC emissions was associated with the addition of rubber to asphalt. Differently, the low heterogeneity (*p* = 0.81, I^2^ = 0%) and high meta-RR of 8.44 (95% CI 2.90 to 24.56) indicated that an obvious increase in benzothiazole mission was associated with the addition of rubber to asphalt (Figure 11). The above results demonstrate that DRMA hardly increases the total gas emissions compared with asphalt but significantly promotes the benzothiazole mission, which should provide guidance for the application of DRMA in pavement construction.

In summary, pavement based on DRMA not only provides improved performance throughout its service life but also delivers significant benefits in terms of human health, ecosystem protection and minimizing resource depletion.

### 5.3. Economic Effects

In general, rubber has certain direct and indirect economic benefits. It extends the structural life and surface life of the pavement, reduces the consumption of capital, materials, labor and energy during the whole life cycle and decreases the number of major repairs and surface maintenance. Importantly, it realizes the resourceful reuse of a large number of waste tires, greatly reducing the pressure of “black pollution”, with huge indirect benefits. However, it should be noticed that rubber asphalt technology differs from traditional SBS-modified asphalt technology in that rubber asphalt technology is slightly more complex than traditional SBS-modified asphalt technology, which may lead to increased equipment acquisition costs as well as maintenance costs.

For a heavy-duty road, though the use of DRMA increases the costs of the lower pavement layer and synchronous macadam seal, it reduces the cost of the upper pavement layer and avoids the construction of the prime coat, tack coat and flexible base. Overall, DRMA can reduce the cost of pavement structures by 10–20%. Other direct and indirect benefits are not discussed here.

### 5.4. Applications

Rubber-modified asphalt has been studied in China for almost 50 years, and in recent years, the technology has been widely used in practical engineering.

With the rapid development of highways, rubber asphalt production technology has gone through several stages. The traditional wet process involves adding finely ground GTR to hot asphalt and mixing and homogenizing the system into rubber asphalt. The average particle size of GTR used in this process is ca. 0.56 mm. Due to the cross-linking network structure, the process requires a high temperature, pollutes the environment and increases energy consumption. To change this situation, terminal blending technology has been developed, which makes it possible to improve compatibility and produce rubber asphalt on a large scale in plants [37,38]. However, it seems that this technology still suffers from harmful gas emissions and requires a high asphalt-to-aggregate ratio. The technology of environmentally modifying asphalt with DR can save the production time of rubber asphalt and greatly avoid segregation, reduce emissions and lower the asphalt-to-aggregate ratio, with a similar preparation condition to the mature process of SBS-modified asphalt. And the obtained DRMA shows improved properties as reported [39].

DRMA can be applied to different structural layers of the road. For example, layers for the Jiuquan-Jiayuguan Ring Expressway in Gansu province from bottom to top are: rubber asphalt stress-absorbing layer, high-content DRMA ATB-25 lower pavement layer and viscoelastic DRMA SMA-13 upper pavement layer. The combined structural layer based on DRMA brings excellent resistance to cracking, shear and aging and is expected to double the service life.

## 6. Conclusions

The covalently cross-linked tire rubber presents an extreme challenge in reusing waste rubbers in roads. DRMA with a finely dispersed or colloidal structure has been regarded as a potential binder used as road material because of the improved properties in terms of storage stability, easy processing and high content of incorporation.

This paper analyzed the rubber asphalt colloid. The DR included CB, sol fraction and inorganic species such as ZnO, silica and ZnS. Based on the structure and composites of DR, base asphalt fractions and the mixing mechanism between them were analyzed at different levels. Furthermore, different interactions between DR and asphalt were summarized. Finally, the sustainability of rubber asphalt was studied and evaluated. This discussion proposed a mechanism for forming the colloidal structure of DRMA, which should provide a theoretical basis for preparing rubber asphalt with outstanding performance. The following conclusions can be drawn:

(1) The DR mainly consisting of CB, sol fraction and inorganic species (ZnO, silica, ZnS, etc.) shows improved dispersibility and reinforcement in base asphalt compared with GTR, providing an important foundation for the application of rubber asphalt.

(2) The functional groups including -OH, -NH_2_, -SH, -COOH and -SO_2_- from CB tend to physically/chemically interact with the resins or asphaltenes, as both resins and asphaltenes are full of polar groups. As a result, N, O and S elements show an increase for asphalt after mixing with rubber, which should be responsible for the improved performance of rubber asphalt.

(3) Multiple interactions occurred with the sol fraction, CB, inorganic salts of DR and four fractions of asphalt, including π-π nonbonding intermolecular interactions, hydrogen bonds, electrostatic interactions, covalent interactions and doping of ions into crystal defect.

(4) The soluble sol fraction has similar solubility parameters with asphalt and can be dissolved into asphalt more easily, which significantly improves the compatibility between DR and asphalt. The obtained DRMA is expected to be stable in a colloidal structure, in which the sol fraction/saturates/aromatics are the medium of suspension, CB/inorganic component/asphaltenes are the suspended particles, and resins are the peptizator.

(5) DRMA shows high performance and significant economic benefits. The addition of DR in asphalt hardly contributed to the emission of volatile organic compounds (VOCs) and the release of polycyclic aromatic hydrocarbons (PAHs), and the presence of VOCs and PAHs depends mostly on asphalt. However, a sharp increase in benzothiazole is proved, derived from a DRMA simulated exposure to acid rain.

At present, rubber asphalt shows an attractive prospect in terms of outstanding performance, economic benefits, energy saving, etc. On the other hand, rubber asphalt still significantly increases the emissions of a few harmful substances, although it will not significantly increase the total emissions, which may have a negative impact on the environment and health. In the future, rubber asphalt should be further promoted and extensively used, taking full account of environmental benefits, performance, health effects and other aspects.

## Figures and Tables

**Table 1:** Molecular weight and its distribution of sol fraction [11].

Temperature/°C	${\bar{M}}_{n}$(10^4^ g/mol)	${\bar{M}}_{w}$(10^4^ g/mol)	${\bar{M}}_{w}/{\bar{M}}_{n}$
220	1.19	6.12	5.12
240	1.25	6.01	4.79
260	1.42	6.89	4.84
280	1.34	6.29	4.70
300	0.41	2.28	5.57

**Table 2:** Chemical properties of asphalt and four fractions [34].

Fractions	H/C	C (%)	H (%)	O (%)	N (%)	S (%)	M_n_ (g/mol)
Asphalt	1.5	80–88	8–12	0–2	0–2	0–9	600–1500
Saturates	1.9	78–84	12–14	<0.1	<0.1	<0.1	470–880
Aromatics	1.5	80–86	9–13	0.2	0.4	0–4	570–980
Resins	1.4	67–88	9–12	0.3–2	0.2–1	0.4–5	780–1400
Asphaltenes	1.1	78–88	7–9	0.3–5	0.6–4	0.3–11	800–3500
